# Supplementary material for: Cryptic evolved melts beneath monotonous basaltic shield volcanoes in the Galápagos Archipelago
Source: Nat Commun. 2020 Jul 28;11:3767. doi: 10.1038/s41467-020-17590-x (PMC7387547; doi:10.1038/s41467-020-17590-x)
Supplement: Supplementary file 1 — Supplementary Information [file 41467_2020_17590_MOESM1_ESM.pdf]

Supplementary Information

Cryptic evolved melts beneath monotonous basaltic shield volcanoes in the Galápagos Archipelago

Michael J. Stock[*1,2], Dennis Geist[3,4], David A. Neave[5], Matthew L.M. Gleeson[2], Benjamin Bernard[6], Keith A. Howard[7], Iris Buisman[2], John Maclennan[2]

[1] Department of Geology, Trinity College Dublin, Dublin, Ireland

[2] Department of Earth Sciences, University of Cambridge, Cambridge, UK

[3] Department of Geology, Colgate University, Hamilton, NY, USA

[4] Division of Earth Sciences, U.S. National Science Foundation, Alexandria, VA, USA

[5] Department of Earth and Environmental Sciences, The University of Manchester, Manchester, UK

[6] Instituto Geofísico, Escuela Politécnica Nacional, Quito, Ecuador

[7] United States Geological Survey, Menlo Park, CA, USA

[*]E-mail: Michael.Stock@tcd.ie

Supplementary Note 1

Supplementary Note 2

Supplementary Note 3

Supplementary Tables

Supplementary Figures

Supplementary References

Supplementary Note 1

Chronology of the 2015 Wolf eruption

The edifice of Wolf volcano is resurfaced by new lava flows every few thousand years[1]; eight eruptions occurred in the last century[2,3]. After 33 years of quiescence, eyewitnesses report that the 2015 eruption of Wolf volcano began with an explosion from a circumferential fissure on the southeast side of the caldera between 00:30 and 00:45 (all timings are local time; GMT-6) on 25 May, which coincided with the last and largest in a series of pre-eruptive seismic events recorded by an Instituto Geofisico seismometer on Fernandina (FER1; recorded at 00:58)[2]. The explosion produced reticulitic tephra that was deposited on the flanks of the edifice and carried >1,400 km eastwards to the Ecuadorian mainland[4]. The emission rate subsequently decreased, and the circumferential fissure continued to produce lava flows that first moved southeast, then eastwards, reaching the coast between 26 and 27 May (Fig. 1b). Activity from the circumferential fissure decreased from 31 May but reinitiated from a new vent on the south side of the caldera on 13 June, producing caldera-fill lavas until the eruption ended on 30 June (Fig. 1b). In total, the eruption produced ~$87 \cdot 10^6$ m^3 of basaltic lava, making it the fourth largest Galápagos eruption in the past 40 years[2].

The 2015 eruption was preceded by a prolonged period of surface inflation, related to a shallow magma source centred within the caldera, and was accompanied by edifice-wide deflation, related to magma withdrawal from a deeper source[5]. Before the eruption, Wolf was not underlain by a fully transcrustal magmatic system, but rather at least two discrete storage regions in the upper (~1.1 km depth) and lower crust (>6.1–8.8 km depth)[6].

Chronology of the 1968 Fernandina eruption

Fernandina is currently the most frequently active Galápagos volcano, with the entire edifice resurfaced on timescales <4.3 kyr; at least 20 eruptions occurred in the last century[3,7]. The 1968 Fernandina eruption is the only recent Galápagos eruption to have produced significant explosive activity and was accompanied by the largest mafic caldera collapse since the advent of modern volcano monitoring (2 km^3; ref. [8]). Following seven years of repose, the 1968 eruption of Fernandina volcano commenced from a radial fissure at ~15:00 on 21 May, producing lava flows that covered ~10 km^2 (ref. [8]). The fissure eruption ended by 25 May but periodic earthquakes continued[9]. On 11 June, a vapour cloud was observed above the island at ~10:16 and the main phase of the eruption began with a major hydromagmatic paroxysm at 17:08, driven by magma interacting with ground water and an intra-caldera lake[8,10]. The explosion originated from an alcove on the western side of the summit caldera, enlarging it and forming a new maar structure[10]. It produced ballistic ejecta close to the vent and a >15 km high plume that deposited pyroclastic material (including juvenile basaltic tephra, plutonic nodules and lithics) on the western flank of the volcano (Fig. 1c)[8,10,11]. Distal ashfall stopped on 12 June and the eruption lasted <7 days[8]. The onset of caldera collapse coincided with the paroxysm and continued for ~10 days[8,9]. The amount of juvenile material ejected in the eruption is significantly smaller than the added caldera volume, likely reflecting either substantial sub-surface magma drainage or discharge from a submarine vent[10].

Like Wolf, geophysical observations from Fernandina indicate at least two discrete magma storage regions: repose periods are characterised by prolonged inflation within the summit caldera, related to a shallow storage zone (~1 km depth), whereas eruptions and major dyke intrusions are accompanied by edifice-wide deflation, caused by magma extraction from a lower crustal storage region (~5 km depth)[12,13].

Supplementary Note 2

Published whole-rock and glass data used in Figs. 2, 9 and Supplementary Figs. 1, 2 are from:

- Allan and Simkin[14]
- Arevalo Jr and McDonough[15]
- Geist et al.[1,16-20]
- Handley et al.[21]
- Koleszar et al.[22]
- Kurz and Geist[23]
- McBirney and Williams[24]
- McBirney et al.[25]
- Naumann et al.[26]
- Peterson et al.[27]
- Reynolds and Geist[28]
- Saal et al.[29]
- Standish et al.[30]
- Teasdale et al.[31]
- White et al.[32]

Supplementary Note 3

X-ray fluorescence spectroscopy (XRF) preparation and analytical methods

Bulk major and trace element concentrations were measured by XRF in 18 lava samples from the 2015 Wolf eruption. Samples were cut into approximately fist-sized sized (~300 cm^3) blocks, removing any oxidised surfaces, and a steel dremel was used to remove any material deposited within vesicles. They were then washed in a distilled water ultrasonic bath to remove any sea spray deposits, before being crushed in a steel jaw crusher at the University of Cambridge and ground to a fine powder in a tungsten-carbide GYRO mill at the University of Edinburgh. Samples were prepared as fused glass discs and pressed powder pellets for major and trace element analysis, respectively, with full preparation details provided by Passmore et al.[33]. Analyses were performed using a Philips PW 2404 instrument at the University of Edinburgh, following the procedures of Fitton et al.[34] with modifications by Fitton and Godard[35].

Electron microprobe analysis (EPMA) analytical methods

Mineral compositions were measured by EPMA using Cameca SX100 and Cameca SXFive instruments in the Departments of Earth Sciences at the University of Cambridge (UK) and Syracuse University (USA), respectively. Wolf samples were analysed on the Cambridge instrument at 15 kV, with a 10 nA, defocussed (5 µm) beam for feldspar and a 40 nA, focussed beam for olivine. Fernandina samples were analysed on the Syracuse instrument with a 15 kV, 20 nA focussed beam for all phases, to facilitate analysis of small crystals. Typical peak count times were 10–20 s for major elements and 60–150 s for minor elements, with the total background count times equalling the on-peak time for each element. Primary calibrations were undertaken using a range of appropriate mineral and metal standards and analyses were internally calibrated using appropriate Smithsonian Microbeam Standards[36] to ensure consistency between analytical sessions. Most analyses returned totals of between 97.5 wt% (96.5 wt% in clinopyroxene) and 101.5 wt%. Data were discarded if they had totals outside of this range or had anomalous major element compositions, suggesting accidental analysis of other phases.

Supplementary Tables

	Wolf		
	Evolved end-member (Rhyolite-MELTS; 300 MPa)	Primitive end-member #1 (W9562)[1]	Primitive end-member #2 (D4A)[1]
**SiO_2**	55.41	48.61	48.66
**TiO_2**	1.85	1.61	4.14
**Al_2O_3**	14.94	17.23	13.39
**FeOt**	8.56	7.79	13.61
**CaO**	6.06	12.74	9.61
**MgO**	2.32	9.50	5.06
**MnO**	0.92	0.13	0.20
**K_2O**	1.78	0.25	0.77
**Na_2O**	5.67	2.16	3.54
**P_2O_5**	1.14	0.19	0.50
**Total**	98.64	100.21	99.48
**K_2O/TiO_2**	0.96	0.16	0.19
**$Mg\#_{liq}$**	36.22	71.89	43.81
	**Fernandina**		
	**Evolved end-member (Rhyolite-MELTS; 500 MPa)**	**Primitive end-member #1 (D25C-2-34)[22]**	**Primitive end-member #2 (D30A)[17]**
**SiO_2**	60.35	48.11	46.56
**TiO_2**	1.36	2.46	3.47
**Al_2O_3**	13.65	15.35	12.29
**FeOt**	6.97	8.96	15.40
**CaO**	4.43	12.13	10.50
**MgO**	1.08	8.82	5.17
**MnO**	1.21	0.15	0.21
**K_2O**	2.71	0.32	0.70
**Na_2O**	5.90	2.34	5.12
**P_2O_5**	0.82	0.39	0.49
**Total**	98.47	99.03	99.91
**K_2O/TiO_2**	1.99	0.13	0.20
**$Mg\#_{liq}$**	24.48	67.37	41.32

Supplementary Table 1: End-member liquid compositions used in the Wolf and Fernandina mixing models (Fig. 9). The evolved end-members are from Rhyolite-MELTS (this study) and the primitive end-members are whole-rock and glass compositions from the literature[1,17,22].

Supplementary Figures

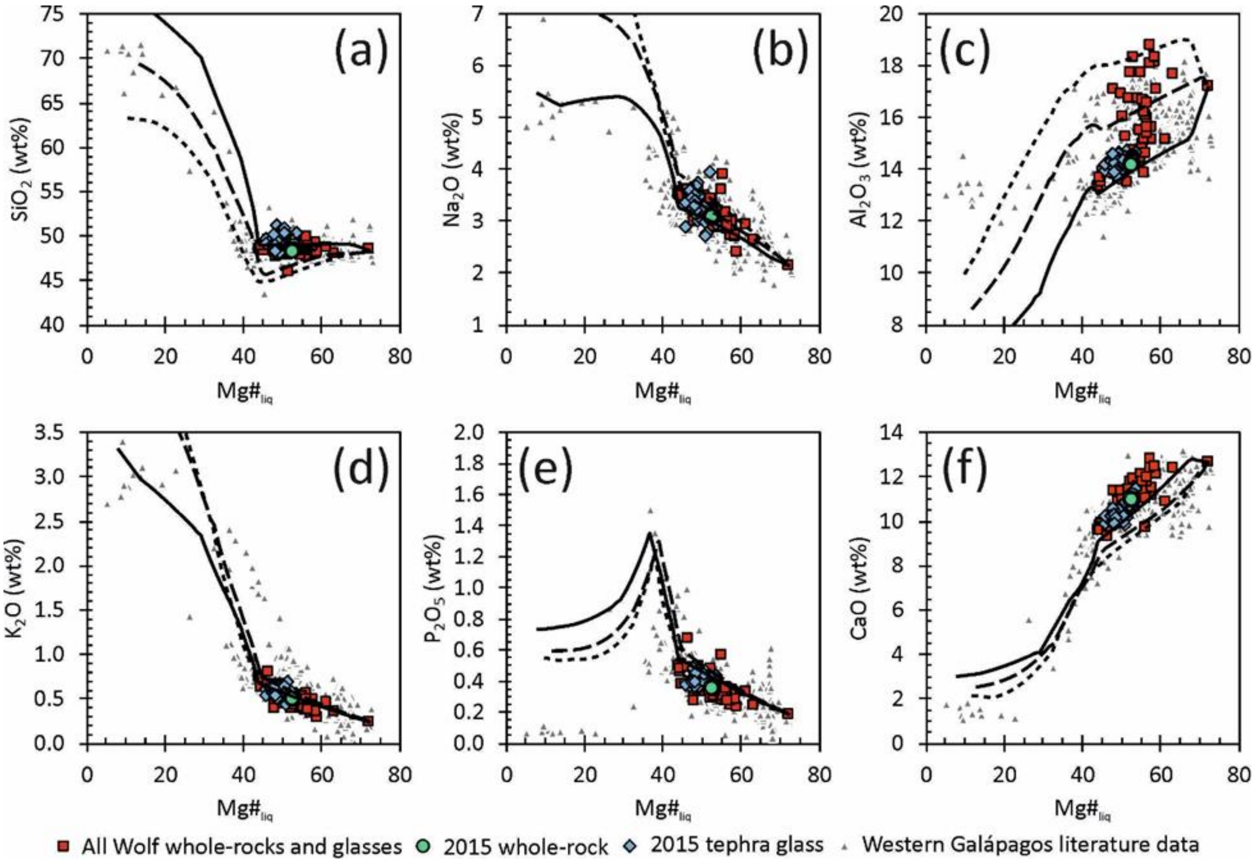

Supplementary Figure 1: Major and minor elements vs. $Mg\#_{liq}$ in liquids from Wolf volcano. The points show: whole-rock, tephra glass, submarine glass and melt inclusion literature data from all volcanoes in the western Galápagos Archipelago (excluding intrusive rocks and plagioclase-ultraphyric lavas); all previously analysed liquids (i.e. whole-rocks and submarine glasses) from Wolf volcano; and whole-rock and glass data from the 2015 Wolf eruption (see legend). References for previously analysed Wolf liquids and literature data from all western Galápagos volcanoes are in Supplementary Note 2. Glass data for the 2015 Wolf eruption are from Stock et al.[6] and whole-rock data are from this study. Characteristic 2σ analytical uncertainties for our whole-rock analyses are less than the size of a data point. The black lines show liquid lines of descent calculated using Rhyolite-MELTS at 50 MPa (solid line), 300 MPa (dashed line) and 500 MPa (dotted line).

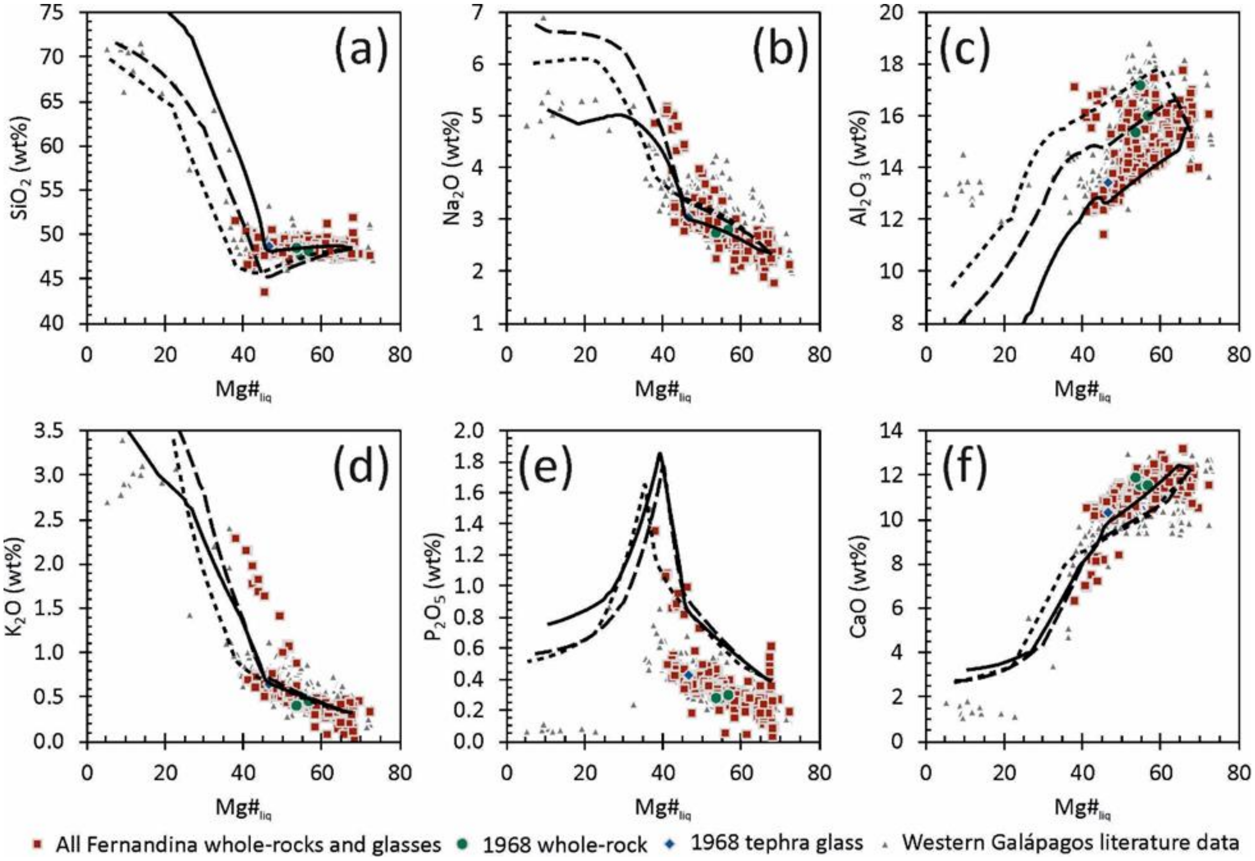

Supplementary Figure 2: Major and minor elements vs. $Mg\#_{liq}$ in liquids from Fernandina. The points show: whole-rock, tephra glass, submarine glass and melt inclusion literature data from all volcanoes in the western Galápagos Archipelago (excluding intrusive rocks and plagioclase-ultraphyric lavas); all previously analysed liquids (i.e. whole-rocks, tephra glasses, submarine glasses and melt inclusions) from Fernandina; and whole-rock and glass data from the 1968 Fernandina eruption (see legend). References for previously analysed Fernandina liquids and literature data from all western Galápagos volcanoes are in Supplementary Note 2. Glass and whole-rock data for the 1968 Fernandina eruption are from Allan and Simkin[14]. The black lines show liquid lines of descent calculated using Rhyolite-MELTS at 50 MPa (solid line), 300 MPa (dashed line) and 500 MPa (dotted line).

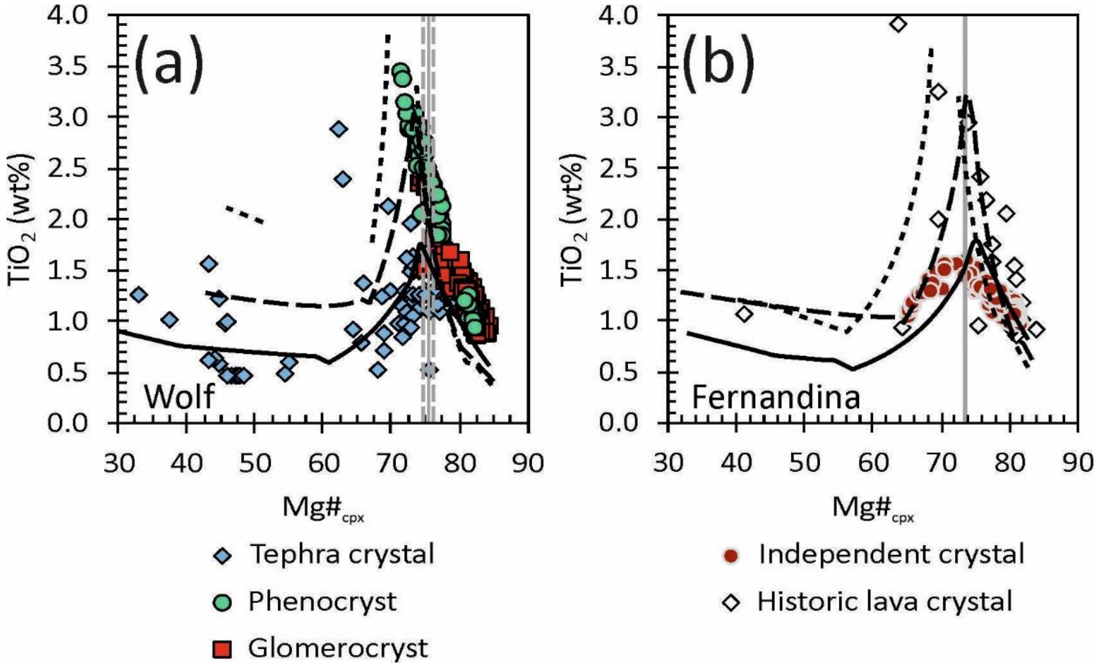

Supplementary Figure 3: Clinopyroxene TiO_2 vs Mg#$_{cpx}$ in **(a)** lava and tephra samples from the 2015 Wolf eruption and **(b)** nodule samples from the 1968 Fernandina eruption and lava samples from historic Fernandina eruptions. Crystals are classified according to their textural association (see legend). Crystal compositions from historic Fernandina lavas are from Allan and Simkin[14]. Characteristic 2σ analytical uncertainties for our clinopyroxene analyses are less than the size of a data point. The grey lines show the compositions of crystals calculated to be in equilibrium with the 2015 Wolf tephra glass (solid lines – average composition; dashed lines – 1σ compositional range)[6] and 1968 Fernandina scoria glass[14], using the model of Putirka[37] at 1160 °C and 1130 °C, respectively (the approximate pre-eruptive crystallisation temperatures)[6,14]. The black lines show the trajectory of clinopyroxene compositional evolution calculated using Rhyolite-MELTS at 50 MPa (solid line), 300 MPa (dashed line) and 500 MPa (dotted line). In the Wolf models, clinopyroxene comes onto the liquidus at 1189 °C (50 MPa), 1234 °C (300 MPa) and 1269 °C (500 MPa). In the Fernandina models, clinopyroxene comes onto the liquidus at 1176 °C (50 MPa), 1222 °C (300 MPa) and 1257 °C (500 MPa).

Supplementary References

1 Geist, D. J. *et al.* Wolf Volcano, Galápagos Archipelago: Melting and magmatic evolution at the margins of a mantle plume. *Journal of Petrology* **46**, 2197-2224 (2005).

2 Bernard, B. *et al.* Chronology and phenomenology of the 1982 and 2015 Wolf volcano eruptions, Galápagos Archipelago. *Journal of Volcanology and Geothermal Research* **374**, 26-38 (2019).

3 Global Volcanism Program. *Volcanoes of the World, v. 4.8.3*. (ed Venzke, E.) (Smithsonian Institution, 2013). Downloaded 31 Oct 2019. https://doi.org/10.5479/si.GVP.VOTW4-2013

4 Bernard, B. *et al.* Preliminary results on the 2015 eruption of Wolf Volcano, Isabela Island, Galápagos: chronology, dispersion of the volcanic products, and insight into the eruptive dynamics. *AGU Fall Meeting 2015*, V31B-3022 (2015).

5 Xu, W., Jónsson, S., Ruch, J. & Aoki, Y. The 2015 Wolf volcano (Galápagos) eruption studied using Sentinel-1 and ALOS-2 data. *Geophysical Research Letters* **43**, 9573-9580 (2016).

6 Stock, M. J. *et al.* Integrated petrological and geophysical constraints on magma system architecture in the western Galápagos Archipelago: insights from Wolf volcano. *Geochemistry, Geophysics, Geosystems* **19**, 4722-4743 (2018).

7 Kurz, M. D., Rowland, S., Curtice, J., Saal, A. & Naumann, T. Eruption rates for Fernandina volcano: a new chronology at the Galápagos hotspot center. In *The Galapagos: A natural laboratory for the Earth sciences* (eds Harpp, K. S., Mittelstaedt, E., d'Ozouville, N., & Graham, D. W.), *Ch*. 4, 41-54 (American Geophysical Union, Washington, DC, 2014).

8 Simkin, T. & Howard, K. A. Caldera Collapse in the Galapagos Islands, 1968: The largest known collapse since 1912 followed a flank eruption and explosive volcanism within the caldera. *Science* **169**, 429-437 (1970).

9 Filson, J., Simkin, T. & Leu, L. k. Seismicity of a caldera collapse: Galapagos Islands 1968. *Journal of Geophysical Research* **78**, 8591-8622 (1973).

10 Howard, K. A., Simkin, T., Geist, D. J., Merlen, G. & Nolf, B. Large hydromagmatic eruption related to Fernandina Volcano's 1968 caldera collapse—Deposits, landforms, and ecosystem recovery. In *Field Volcanology: A Tribute to the Distinguished Career of Don Swanson, Special Paper 538* (eds Poland, M., Garcia, M. O., Camp, V. E., & Grunder, A.), 385-408 (Geological Society of America, Boulder, CO, 2018).

11 Simkin, T. Geology of Galapagos Islands. *Biological Journal of the Linnean Society* **21**, 61-75 (1984).

12 Geist, D., Chadwick, W. & Johnson, D. Results from new GPS and gravity monitoring networks at Fernandina and Sierra Negra Volcanoes, Galápagos, 2000–2002. *Journal of Volcanology and Geothermal Research* **150**, 79-97 (2006).

13 Bagnardi, M. & Amelung, F. Space-geodetic evidence for multiple magma reservoirs and subvolcanic lateral intrusions at Fernandina Volcano, Galápagos Islands. *Journal of Geophysical Research: Solid Earth* **117**, B10406 (2012).

14 Allan, J. F. & Simkin, T. Fernandina Volcano's evolved, well-mixed basalts: Mineralogical and petrological constraints on the nature of the Galápagos plume. *Journal of Geophysical Research: Solid Earth* **105**, 6017-6041 (2000).

15 Arevalo Jr, R. & McDonough, W. F. Tungsten geochemistry and implications for understanding the Earth's interior. *Earth and Planetary Science Letters* **272**, 656-665 (2008).

16 Geist, D., Howard, K. A. & Larson, P. The generation of oceanic rhyolites by crystal fractionation: the basalt-rhyolite association at Volcán Alcedo, Galápagos Archipelago. *Journal of Petrology* **36**, 965-982 (1995).
17 Geist, D. J. *et al.* Submarine Fernandina: Magmatism at the leading edge of the Galápagos hot spot. *Geochemistry, Geophysics, Geosystems* **7**, Q12007 (2006).
18 Geist, D. J. *et al.* The 2005 eruption of Sierra Negra volcano, Galápagos, Ecuador. *Bulletin of Volcanology* **70**, 655-673 (2008).
19 Geist, D. *et al.* Construction of the Galápagos platform by large submarine volcanic terraces. *Geochemistry, Geophysics, Geosystems* **9**, Q03015 (2008).
20 Geist, D. *et al.* Volcanic evolution in the Galápagos: The dissected shield of Volcan Ecuador. *Geochemistry, Geophysics, Geosystems* **3**, 1061 (2002).
21 Handley, H. K., Turner, S., Berlo, K., Beier, C. & Saal, A. E. Insights into the Galápagos plume from uranium-series isotopes of recently erupted basalts. *Geochemistry, Geophysics, Geosystems* **12**, Q0AC14 (2011).
22 Koleszar, A. *et al.* The volatile contents of the Galapagos plume; evidence for H_2O and F open system behavior in melt inclusions. *Earth and Planetary Science Letters* **287**, 442-452 (2009).
23 Kurz, M. D. & Geist, D. Dynamics of the Galapagos hotspot from helium isotope geochemistry. *Geochimica et Cosmochimica Acta* **63**, 4139-4156 (1999).
24 McBirney, A. R. & Williams, H. *Geology and petrology of the Galapagos Islands, Memoir 118*. (Geological Society of America, Boulder, CO, 1969).
25 McBirney, A. *et al.* The Galapagos volcano Alcedo: a unique ocean caldera. *Journal of Volcanology and Geothermal Research* **26**, 173-177 (1985).
26 Naumann, T., Geist, D. & Kurz, M. Petrology and geochemistry of Volcan Cerro Azul: Petrologic diversity among the western Galápagos volcanoes. *Journal of Petrology* **43**, 859-883 (2002).
27 Peterson, M. *et al.* Submarine basaltic glasses from the Galapagos Archipelago: determining the volatile budget of the mantle plume. *Journal of Petrology* **58**, 1419-1450 (2017).
28 Reynolds, R. W. & Geist, D. J. Petrology of lavas from Sierra Negra volcano, Isabela Island, Galápagos archipelago. *Journal of Geophysical Research: Solid Earth* **100**, 24537-24553 (1995).
29 Saal, A. *et al.* The role of lithospheric gabbros on the composition of Galapagos lavas. *Earth and Planetary Science Letters* **257**, 391-406 (2007).
30 Standish, J., Geist, D., Harpp, K. & Kurz, M. D. The emergence of a Galápagos shield volcano, Roca Redonda. *Contributions to Mineralogy and Petrology* **133**, 136-148 (1998).
31 Teasdale, R., Geist, D., Kurz, M. & Harpp, K. 1998 Eruption at Volcán Cerro Azul, Galápagos Islands: I. Syn-Eruptive Petrogenesis. *Bulletin of Volcanology* **67**, 170-185 (2005).
32 White, W. M., McBirney, A. R. & Duncan, R. A. Petrology and geochemistry of the Galápagos Islands: Portrait of a pathological mantle plume. *Journal of Geophysical Research: Solid Earth* **98**, 19533-19563 (1993).
33 Passmore, E., Maclennan, J., Fitton, G. & Thordarson, T. Mush disaggregation in basaltic magma chambers: evidence from the AD 1783 Laki eruption. *Journal of Petrology* **53**, 2593-2623 (2012).

34 Fitton, J., Saunders, A., Larsen, L., Hardarson, B. & Norry, M. Volcanic rocks from the southeast Greenland margin at 63°N: Composition, petrogenesis and mantle sources. In *Proceedings of the Ocean Drilling Program, Scientific Results 152* (eds Saunders, A., Larsen, H. C., & Wise, S. W.), 331-350 (College Station, TX, 1998).
35 Fitton, J. G. & Godard, M. Origin and evolution of magmas on the Ontong Java Plateau. *Geological Society, London, Special Publications* **229**, 151-178 (2004).
36 Jarosewich, E., Nelen, J. A. & Norberg, J. A. Reference samples for electron microprobe analysis. *Geostandards and Geoanalytical Research* **4**, 43-47 (1980).
37 Putirka, K. D. Thermometers and barometers for volcanic systems. *Reviews in Mineralogy and Geochemistry* **69**, 61-120 (2008).
