# Supplementary material for: Cryptic evolved melts beneath monotonous basaltic shield volcanoes in the Galápagos Archipelago
Source: Nat Commun. 2020 Jul 28;11:3767. doi: 10.1038/s41467-020-17590-x (PMC7387547; doi:10.1038/s41467-020-17590-x)
Supplement: Supplementary file 3 — Descriptions of Additional Supplementary Files [file 41467_2020_17590_MOESM3_ESM.pdf]

Descriptions of Additional Supplementary Files

Supplementary Dataset 1

Description: Whole-rock major and trace element compositions of Wolf lavas erupted in 2015.

Supplementary Dataset 2

Description: Plagioclase compositions from the 2015 eruption of Wolf volcano. Data are separated into phenocrysts from lava samples, glomerocrysts from lava samples, synneusis aggregates from lava samples and crystals from tephra samples.

Supplementary Dataset 3

Description: Olivine compositions from the 2015 eruption of Wolf volcano. Data are separated into phenocrysts from lava samples, glomerocrysts from lava samples and crystals from tephra samples.

Supplementary Dataset 4

Description: Plagioclase compositions from nodules produced during the 1968 eruption of Fernandina volcano. Data are separated into independent grains surrounded by matix material, inclusions within other crystals and crystals bounding miarolitic cavities. MgO may be below detection limit (~0.018 wt%) but is included for comparison.

Supplementary Dataset 5

Description: Olivine compositions from nodules produced during the 1968 eruption of Fernandina volcano.

Supplementary Dataset 6

Description: Clinopyroxene compositions from nodules produced during the 1968 eruption of Fernandina volcano.

Supplementary Dataset 7

Description: Modelled liquid and plagioclase compositions during cooling and fractional crystallisation at Wolf (starting liquid - W9562) and Fernandina (starting liquid - D25C-2-34) volcanoes. Liquid and plagioclase major element compositions were calculated using Rhyolite-MELTS. Plagioclase Ti concentrations were determined using Rhyolite-MELTS ouputs and DTi values calcuated after Nielsen et al. (2017).
